# Post-stroke Delivery of Valproic Acid Promotes Functional Recovery and Differentially Modifies Responses of Peri-Infarct Microglia

**DOI:** 10.3389/fnmol.2021.639145

**Published:** 2021-05-28

**Authors:** Tung-Tai Kuo, Vicki Wang, Jui-Sheng Wu, Yuan-Hao Chen, Kuan-Yin Tseng

**Affiliations:** ^1^Department of Neurological Surgery, Tri-Service General Hospital, Taipei, Taiwan; ^2^Graduate Institute of Medical Sciences, National Defense Medical Center, Taipei, Taiwan; ^3^Department of Biology and Anatomy, National Defense Medical Center, Taipei, Taiwan; ^4^Department of Neurological Surgery, Tri-Service General Hospital, National Defense Medical Center, Taipei, Taiwan

**Keywords:** distal middle cerebral artery occlusion (dMCAO), valproic acid (VPA), microglia activation, galectin-3 (Gal-3), ischemic stroke

## Abstract

The specific role of peri-infarct microglia and the timing of its morphological changes following ischemic stroke are not well understood. Valproic acid (VPA) can protect against ischemic damage and promote recovery. In this study, we first determined whether a single dose of VPA after stroke could decrease infarction area or improve functional recovery. Next, we investigated the number and morphological characteristic of peri-infarct microglia at different time points and elucidated the mechanism of microglial response by VPA treatment. Male Sprague-Dawley rats were subjected to distal middle cerebral artery occlusion (dMCAo) for 90 min, followed by reperfusion. Some received a single injection of VPA (200 mg/kg) 90 min after the induction of ischemia, while vehicle-treated animals underwent the same procedure with physiological saline. Infarction volume was calculated at 48 h after reperfusion, and neurological symptoms were evaluated. VPA didn’t significantly reduce infarct volume but did ameliorate neurological deficit at least partially compared with vehicle. Meanwhile, VPA reduced dMCAo-induced elevation of IL-6 at 24 h post-stroke and significantly decreased the number of CD11b-positive microglia within peri-infarct cortex at 7 days. Morphological analysis revealed that VPA therapy leads to higher fractal dimensions, smaller soma size and lower circularity index of CD11b-positive cells within peri-infarct cortex at both 2 and 7 days, suggesting that VPA has core effects on microglial morphology. The modulation of microglia morphology caused by VPA might involve HDAC inhibition-mediated suppression of galectin-3 production. Furthermore, qPCR analysis of CD11b-positive cells at 3 days post-stroke suggested that VPA could partially enhance M2 subset polarization of microglia in peri-infarct cortex. Analysis of VPA-induced changes to gene expressions at 3 days post-stroke implies that these alternations of the biomarkers and microglial responses are implicated in the upregulation of wound healing, collagen trimmer, and extracellular matrix genes within peri-infarct cortex. Our results are the first to show that a low dose of VPA promotes short-term functional recovery but does not alter infarct volume. The decreases in the expression of both IL-6 and galectin-3 might influence the morphological characteristics and transcriptional profiles of microglia and extracellular matrix remodeling, which could contribute to the improved recovery.

## Introduction

Stroke is one of the leading causes of death and adult disability worldwide (Feigin et al., 2015). Primary ischemic stroke causes the death of neurons, astrocytes, and oligodendrocytes, as well as blood capillary damage. This destruction results in a series of pathological reactions known as secondary injuries, including blood barrier disruption and inflammatory responses (Wang et al., 2011). Then, various types of inflammatory cells, such as microglia, T cells, and macrophages, are recruited to the lesion to remove cell debris (Wang et al., 2019). However, these inflammatory cells cause secondary cell death, expansion of the lesion region, and functional impairment of the body by elicit excessive production of cytokines and chemokines (Lambertsen et al., 2012; Lu et al., 2013). Most of the studies were concentrated on the neuroprotective property; thence, there is needed to identify drug targets or develop a new therapeutic way that would accelerate the recovery after ischemic brain injury by addressing postischemic pathologic mechanisms such as neuroinflammation (Anttila et al., 2018). A viable therapeutic strategy that modulates the brain inflammatory responses afterward stroke is recognized to boost functional recovery from ischemic stroke. However, few studies have taken this approach, and little is known about how recovery from stroke relates to neuroinflammation (Lakhan et al., 2009; Ahmad and Graham, 2010).

Valproic acid (VPA), a histone deacetylase (HDAC) inhibitor, is widely used for the treatment of migraine and epileptic seizures and as a mood stabilizer in treating bipolar disorder (Chateauvieux et al., 2010). *In vitro*, treatment with VPA suppresses lipopolysaccharide-induced production of TNF-α and IL-6, attenuates glutamate-induced excitotoxicity, and inhibits ischemia-induced fast sodium and high-voltage-activated calcium currents (Suda et al., 2013; Mairuae and Cheepsunthorn, 2018). In rat models of transient ischemia, VPA exhibits anti-inflammatory and neuroprotective effects through the inhibition of HDAC activity and induction of HSP70, which attenuates ischemia-reperfusion injury. In addition, it ameliorates blood-brain barrier (BBB) disruption through inhibition of the nuclear translocation of nuclear factor-κB activation and matrix metalloproteinase 9 production (Ren et al., 2004; Wang et al., 2011; Suda et al., 2013). Furthermore, delayed VPA treatment could enhance white matter repair and neurogenesis in ischemic brain (Liu et al., 2012). In an animal model of spinal cord injury, VPA was further shown to decrease purinergic P2 × 4 receptor expression in activated microglia, as well as to ameliorate microgliosis (Masuch et al., 2016; Chen et al., 2018). Although the studies referenced above suggested that VPA therapy is involved in microglial activation, those investigations determined activated microglia based only on cellular density in brain sections immunolabeled to detect either ionized calcium binding adaptor molecule 1 (Iba1) or CD11b (Xuan et al., 2012). There have been no studies, however, aimed at better characterizing the morphological changes and polarized phenotype of peri-infarct microglia after VPA administration. Meanwhile, VPA therapy has been demonstrated to cause dose-dependent side effects, like depression, ataxia, arrhythmia, and rare but fatal hepatotoxicity (Anderson et al., 1992; Isoherranen et al., 2003). Relatively high doses of VPA of 300 mg/kg/day and above were shown to induce hepatotoxicity and neurological side effects in animal studies (Tai et al., 2014). Thus, we focused to study if the post-stroke injection of a lower dose of VPA, 200 mg/kg, in rats would promote recovery and if this recovery, if any, is implicated in altered biomarkers, cytokines, and transcriptional profiles within peri-infarct cortex. Furthermore, we quantitated the subtleties of microglial morphology with fractal analysis, soma size and circularity index following cortical ischemia-reperfusion insult because morphology has not yet been well studied. As microglia show obviously morphological changes on day 2 post-stroke in the peri-infarction cortex, we administered VPA after the onset of reperfusion. Here, we show how post-stroke of VPA administration, 200 mg/kg, promoted behavioral recovery during the short 2-day testing period, accompanied by modulating microglia/macrophage activation, and further explore the underlying molecular mechanisms.

## Materials and Methods

### Animals

The total of 157 Male adult Sprague-Dawley rats (300–350 g; 36 Sham, 23 Sham + VPA, 50 dMCAo + vehicle, 48 dMCAo + VPA), based on previous experience involving similar experiment settings, were used for this study. Those animals were housed at the National Defense Medical Center’s Animal Center with a 12-h light/dark cycle, temperature of 25 ± 2°C, 55% humidity, 2–4 animals per cage, and *ad libitum* standard diet and water. The experimental protocol was approved by the Institutional Animal Care and Use Committee (IACUC; protocol number 16-258) of the National Defense Medical Center, Taiwan, R.O.C., which is accredited by the Association for Assessment and Accreditation of Laboratory Animal Care International (AAALAC International). All experiments were performed in a blinded manner, and the experimental results are reported according to the ARRIVE guidelines.

### Distal Middle Cerebral Artery Occlusion Model and VPA Treatment

A cortical stroke was induced in each rat by occluding the distal middle cerebral artery (dMCA) along with bilateral common carotid arteries (CCAs) (Chen et al., 1986). Each rat was anesthetized with 4% chloral hydrate (Sigma Aldrich, St. Louis, MO, United States) injected intraperitoneally (i.p.; 400 mg/kg), and lidocaine was used as a local anesthetic. The surgery was conducted as described earlier (Airavaara et al., 2010, 2009; Matlik et al., 2014). Briefly, the CCAs were separated through a cervical dissection. A small craniotomy was undertaken on the right side of the skull, and the right dMCA was ligated directly with a 10-0 suture. The CCAs were simultaneously occluded with non-traumatic arterial clips. After 90 min of ischemia, the suture around the MCA and the arterial clips were removed for restoration of blood flow (reperfusion). The body temperature of each rat was maintained at 37°C during the procedures until recovery from anesthesia, when the rat was returned to its home cage. A dose of 200 mg/kg of VPA or normal saline as a vehicle control was injected intraperitoneally into each rat, with the one injection made immediately after the onset of reperfusion. VPA was purchased from Sigma (MO, USA) and dissolved in physiologic saline (Xuan et al., 2012). 2,3,5-triphenyltetrazolium chloride (TTC) staining was used to quantified the infarction volume from 2-mm brain slices at day 2 post-stroke as described previously (Airavaara et al., 2010).

### Behavioral Tests

A body asymmetry test was performed as described previously (Airavaara et al., 2010). First, lifting the rats above the testing table by the tails, and then to count the frequency of initial turnings of the head or upper body contralateral to the ischemic side. This trial was repeated 20 times in total. The modified Bederson’s score was used to assessment the neurological deficits of all rats (Anttila et al., 2018). The cylinder test was carried out as described previously (Runeberg-Roos et al., 2016). The rats were placed inside a clear plastic cylinder (diameter: 35 cm) for 5 min. After raising back on the hind limbs, count the number of first front paw touches to the inner wall of the tube. Locomotor activity was measured using an infrared activity monitor for 1 h (Med Associates, St. Albans, VT, United States).

### Assessment of Cytokines

Tissues from a total of 16 rats were used for the cytokine assay by enzyme-linked immunosorbent assay (ELISA). The ipsilateral peri-infarct cortical and hippocampal tissues were collected at 6, 24, and 48 h post-dMCAo. After homogenization in lysis buffer (PRO-PREP^TM^, iNtRON Biotechnology, South Korea) and centrifugation at 15,000 *g* for 30 min, and then collected the supernatants and stored at –80°C. During quantification, the cytokines (TNFα, IL-1β, IL-6, and IL-10) were normalized to 150 μg of protein in the supernatant using a commercial ELISA kit (R&D Systems, Minneapolis, MN, United States) according to the manufacturer’s instructions.

### Histology, Immunostaining of Free-Floating Sections, and Image Acquisition

We utilized pentobarbital (90 mg/kg i.p.) to anesthetize the rats, which then were transcardially perfusion with 200 ml saline followed by 500 ml of 4% paraformaldehyde. Brains were dehydrated in 30% sucrose at 4°C and using a Leica CM3050 Cryostat to section coronally into 40-μm-thick slices. Sections were taken from 2.1 to –1.0 mm (striatum) relative to bregma, then stored in cryopreservant for storage (20% glycerol, 2% DMSO in 1XPBS). Sections were blocked with 4% BSA (Sigma-Aldrich) + 0.1% Triton X-100 (Sigma-Aldrich), then incubated with primary antibody (rabbit anti-CD11b 1:1000; Abcam) overnight at 4°C. The next day, sections were stained with secondary antibody, followed by incubation with secondary antibodies conjugated with Alexa Fluor^®^ 488 or 568 (1:500). Digital imagining was carried out on an Olympus AX-80 microscope and attached DP-70 digital camera (Olympus America Inc., Center Valley, PA, United States) using a 40x objective. Three coronal brain sections per animal (between bregma −0.45 and −1.85) were imaged in the right hemisphere at each time point (sham, 1, 2, and 7 days post-dMCAo) (Morrison et al., 2017). The regions imaged were in the medial peri-infarct cortex extending 400 μm from the infarct border and in the dorsal striatum underlying the infarct. Therefore, the imaging yielded six digital photomicrographs per animal for analysis.

### Unbiased Stereological Counting of CD11b-Positive Cells in the Peri-Infarct Cortex

CD11b-positive cells in the peri-infarct cortex were counted using unbiased stereology with a stereomicroscope (Olympus BX51) and the StereoInvestigator 6 program (MBF Bioscience) as previously described (Mijatovic et al., 2007). Selected four 40 μm thick coronal slices according to their location relative to the bregma (0.2, 0, –0.26, and –0.4 mm) to obtain results relatively free of bias in the distribution of the cells. This experiment only counted CD11b positive cells with clear microglia morphology. Approximately 80 randomly selected sites were analyzed for per area/slice to ensure accuracy and minimize error.

### Fractal Analysis Using FracLac for ImageJ

For investigating the morphological changes of microglia following ischemic infarct, photomicrographs were acquired from the location (red square in Figures 3A,C,E), 300 μm away from the infarct (peri-infarct cortex), where microglia activation was to be expected high. We utilized our computer-aided morphologic analysis to include fractal analysis (FracLac for ImageJ), which quantifies cell complexity (fractal dimension, fD) (Karperien et al., 2013). Five microglia randomly selected for this analyze within each photomicrograph (6 photomicrographs per animal). A total of 30 cells chosen for fractal analysis (using a grid and random number generator) per animal in each region. The additional structures that abut and surround each cell were eliminated from the analysis by manual deletion using a digitizing tablet and ImageJ. Binary images were then converted to outlines using ImageJ. Fractal dimension (fD) is the assessment of microglia complexity, which quantifies each cell’s contour bounded by the endpoints and process lengths. FracLac for ImageJ calculates the microglia fD for each cell using a box plot protocol that determines the amount of pixel detail with increasing scale, where N = the number of pixels or “detail” at a particular scale (ε) (Figure 5H and Table 1; Morrison et al., 2017). These calculations and relationships were well described in the previous studies (Karperien et al., 2013; Morrison et al., 2017).

**TABLE 1:** Summary of microglia/macrophage morphology measures.

	Measure	Unit	Range	Scale	Sampling	Interpretation
**Fractal dimension**	**Regression slope[*In(N)/In(*?)]**	**DB**	**1-2**	**Individual cell**	**24 cells/animal**	**Cell complexity**
Circularity Index	(4π[area]/[perimeter]2)	CI	0–1	Individual cell	24 cells/animal	Cell circularity

### Manual Analysis of Microglia Morphology

FIJI software (version 2.0) was used for manual morphological analysis (Ferreira et al., 2014; Heindl et al., 2018). In order to prevent overlap with cells located in more superficial or deeper layers, slices containing an identified cells were processed as a maximum intensity projections. Maximum intensity projections of the cells were incepted to create a binary appearance. Quantification of microglia soma was completed using Fiji (Image J) Analyse Particles function with a particle size threshold of 10 pixels, to exclude small pixel nose and extract microglial soma size (green ellipse fit; Figure 5J). Through measurement of the area (white) and the perimeter (red) of a cell from the binary mask, the circularity index (CI) was determined (Figure 5J and Table 1).

### Cell Culture and Treatment

Murine BV-2 microglial cells were gifted from Dr. Mei-Jen Wang (Huang et al., 2016) and maintained in Dulbecco’s modified Eagle’s medium (DMEM) containing 5% fetal bovine serum (FBS), 2 mM L-glutamine, 100 μg/ml streptomycin, and 100 U/ml penicillin (all from Gibco; Thermo Fisher Scientific, Inc., Waltham, MA, United States) at 37°C under humidified 95% O2 and 5% CO2. The cells were then seeded into 24-well plates at a density of 1 × 10^5^/well and maintained at 37°C under humidified 95% O2 and 5% CO2. To detect the expression of acetyl-histone H3, as well as the production of galectin-3 and heat shock protein 70 (HSP70), the medium was replaced with freshly prepared serum-free medium with or without lipopolysaccharide (LPS) (1 μg/ml) and/or a non-toxic dose of VPA (1.5 mM) for 6 h (Mairuae and Cheepsunthorn, 2018).

### Western Blot Analysis

For western blotting, aliquots of the proteins were distributed by electrophoresis on sodium dodecyl sulfate–polyacrylamide gels (8% or 10%) and transferred to a polyvinylidene difluoride membrane. The membranes were rinsed in 0.01 M Tris-buffered saline (pH 7.4) containing 0.1% Triton X-100 for 10 min, blocked in 5% non-fat dry milk for 30 min, and then incubated overnight at 4°C in the presence of acetylated histone H3 antibody against acetylated histone H3 on lys9 (rabbit polyclonal; 1:1000; sigma-Aldrich), HSP70 (rabbit polyclonal; 1:1000; Santa Cruz), galectin-3 (rabbit polyclonal; 1:250; GeneTex), or actin (mouse polyclonal; 1:1000; Abcam). After washing three times with Tris-buffered saline, the membrane was incubated with a horseradish peroxidase-conjugated secondary antibody in Tris-buffered saline containing 5% non-fat dry milk for 2 h at room temperature. Immunoreactivity was detected by enhanced chemiluminescent autoradiography (ECL kit; Amersham Life Science, Arlington Heights, IL, United States). The western blots were captured with a digital camera, and the intensities were quantified with NIH Image J.

### Protein Array Analysis

Brain protein levels were analyzed following dMCAo-induced cortical infarction. Rat brains were collected and homogenized 48 h after dMACo, and the expression levels of 67 proteins were measured using a RayBio^®^ L-Series Rat 67 Antibody Array (RayBiotech, Norcross, GA, United States). Total protein was extracted from 250 mg of cortical tissue with 1 mL of ice-cold tissue protein extraction reagent containing protein degradation inhibitors (Kangcheng, Shanghai, China). Protein concentrations were determined by a BCA Protein Assay Kit (Kangcheng). The RayBio^®^ L-Series Rat 67 Antibody Array membrane (RayBiotech) was blocked for 30 min by adding blocking buffer, and then incubated with the protein samples at room temperature for 2 h. The chip membrane was cleaned with buffer, and then incubated with biotin-labeled antibodies at room temperature for 2 h. After washing with buffer, the membrane was incubated with streptavidin (1:1000) coupled with horseradish peroxidase at room temperature for 2 h. Reacting with chemiluminescence reagent (RayBiotech, Norcross, GA, United States) in the dark and exposing to X-ray film, images were obtained using a film scanner (i3200, Kodak, Rochester, NY, United States). The original biomarker values were first centered and scaled by subtracting the mean of each biomarker from the data and then dividing it by the standard deviation, respectively (Zhou et al., 2017). Centering and scaling results in a uniform mean and scale across all the biomarkers, but leaves their distribution unchanged (Tusher et al., 2001; Yu et al., 2012).

### Analysis of Differential Biomarker Expression

The biomarker values were summarized in terms of mean and standard deviation, or median with minimum and maximum responses across the groups. The fold change between groups was calculated as the ratio of the mean or median. If the biomarkers met or did not meet normality criteria across two groups, the significance of expression difference was evaluated by the paired *t*-test or signed-rank test, respectively. Biomarkers with a *P* adjusted value < 0.05 were considered to be differentially expressed (Tusher et al., 2001).

### RNA Preparation and RNA-Sequencing

Rats were sacrificed on day 3 after MCAo surgery and perfused with 0.9% saline solution before the collection of tissue material (*n* = 4 for vehicle, *n* = 4 for VPA). RNA was extracted from tissues that were taken from the medial peri-infarct cortex on two 1 mm thick sections from positions A/P –0.9 to +0.1 and A/P +1.1 to +2.1. RNA was extracted with Trizol reagent and treated with DNase (#1906, Ambion). RNA purity and quantification were checked using SimpliNano^TM^ - Biochrom Spectrophotometers (Biochrom, MA, United States). RNA degradation and integrity were monitored by Qsep 100 DNA/RNA Analyzer (BiOptic Inc., Taiwan). One μl RNA of each sample was utilized as input material for the RNA sample preparations. Sequencing libraries were set up by means of the KAPA mRNA HyperPrep Kit (KAPA Biosystems, Roche, Basel, Switzerland) following the manufacturer’s recommendations, and index codes were come with attributing sequences to each sample. In a short, captured mRNA was disintegrated by incubating it at a high temperature in the presence of magnesium in KAPA Fragment, Prime and Elute Buffer (1x). First-strand complementary DNA (cDNA) was integrated using random hexamer priming. After converting the cDNA:RNA hybrid into double-stranded cDNA (dscDNA), dUTP was incorporated into the second cDNA strand accompanied by dAMP added to the 3′ ends of the resulting dscDNA. dsDNA adapters with 3′dTMP overhangs were ligated to library insert fragments to generate the library fragments carrying the adapters. For choosing cDNA fragments of preferentially 300∼400 bp in length, the library fragments were purified with the KAPA Pure Beads system (KAPA Biosystems, Roche, Basel, Switzerland). The library carrying appropriate adapter sequences at both ends was amplified using KAPA HiFi HotStart ReadyMix (KAPA Biosystems, Roche, Basel, Switzerland) along with library amplification primers. Lastly, PCR products were purified using the KAPA Pure Beads system, and the library quality was assessed using the Qsep 100 DNA/RNA Analyzer (BiOptic Inc., Taiwan). The RNA-seq data have been deposited in the *RNA-Seq* database at Biotools-rat-RNA prelibrary under accession number BI-ANA-1416.

### Bioinformatics

The original data from high-throughput sequencing (Illumina NovaSeq 6000 platform) was transformed into raw sequenced reads by CASAVA base celling and stored in FASTQ format (Love et al., 2014; Schurch et al., 2016). These data have been submitted to the NCBI BioProject database^1^. The NCBI SRA accession number for these data is PRJNA694497. After cleaning up low-quality reads and eliminating poor-quality bases (Bolger et al., 2014; Kim et al., 2015; Sahraeian et al., 2017), the obtained high-quality data were used for subsequent analysis. Read pairs from each sample were coordinated to the reference genome using the HISAT2 software (v2.1.0) (Kim et al., 2015; Sahraeian et al., 2017). For gene expression, the “Trimmed Mean of M-values” normalization (TMM) as well as “Relative Log Expression” normalization (RLE) was conducted using DEGseq (Wang et al., 2010; Love et al., 2014; Schurch et al., 2016). Differentially expressed genes (DEGs) analysis of two conditions was performed in R using DEGseq (without biological replicate) and DESeq2 (with biological replicate), which are based on the negative binomial distribution and Poisson distribution model, respectively (Anders et al., 2013; Li et al., 2016; Maza, 2016). The resulting *p*-values were adjusted using Benjamini and Hochberg’s approach for controlling the FDR. GO (Kanehisa et al., 2008, 2019) enrichment analysis of DEGs was conducted using clusterProfiler (v3.10.1) (Yu et al., 2012). Gene set enrichment analysis (GSEA) (Subramanian et al., 2005) was performed with 1,000 permutations to identify enriched biological functions and activated pathways from the molecular signatures database (MSigDB). The MSigDB is a collection of annotated gene sets for use with GSEA software, including hallmark gene sets, positional gene sets, curated gene sets, motif gene sets, computational gene sets, GO gene sets, oncogenic gene sets, and immunologic gene sets (Liberzon et al., 2011, 2015). In addition, weighted gene co-expression network analysis (WGCNA) was used to construct the co-expression network based on the correlation coefficient of expression pattern using the WGCNA (v1.64) package in R (Zhang and Horvath, 2005; Langfelder and Horvath, 2008).

### Real-Time Quantitative PCR From Microglia Isolated From the Sham or Stroke Cortex

The microglia harvest was performed as described previously (Anttila et al., 2018). Briefly, the CD11b-positive microglia within the cortex were isolated by magnetic activated cell sorting (MACS). On ice in HBSS without Ca^2+^ and Mg^2+^ dissected the ischemic cortex and the equivalent part of the brains from the sham animals. And then the Neural Tissue Dissociation kit (Miltenyi Biotec) and gentleMACS Dissociator (Miltenyi Biotec) was used to dissociate tissue. Followingly, the cells were suspended in 0.5% BSA in PBS and incubated with Myelin Removal Beads II (1:10, Miltenyi Biotec) for 15 min at 4°C and filtered through a LS column (Miltenyi Biotec) using a Quadro-MACS Separator (Miltenyi Biotec). Lastly, the cells were incubated at 4°C for 10 min with mouse anti-CD11b: FITC antibody (1:10, #MCA275FA, AbD Serotec) (Anttila et al., 2018). After washing along with resuspending in 0.5% BSA with 2 mM EDTA in PBS, cells were incubated with anti-FITC MicroBeads (1:10, Miltenyi Biotec) for 15 min at 4°C. Following resuspension in 0.5% BSA with 2 mM EDTA in PBS, the cell suspension was applied to a LS column placed on a QuadroMACS Separator. Total RNA was then extracted from the gentleMACS-isolated microglia using RNeasy Plus micro/mini kits (Qiagen, Hilden, Germany), according to the manufacturer’s protocol.

RNA quality was assessed by means of an Agilent Bioanalyzer (Agilent, Santa Clara, CA, United States). For the rat samples, cDNA was synthesized by using an oligo-T18 primer and Maxima H minus reverse transcriptase (#EP0751; Thermo Fisher Scientific). Real-time quantitative PCR (qPCR) was performed with Lightcycler^®^480 SYBR Green I Master complemented with 2.5 pmol of primers (Table 2) following a Lightcycler^®^480 real-time PCR system (Roche Diagnostics). Reactions were performed in triplicate and analyzed with Lightcycler^®^480 Software. Using agarose gel electrophoresis to verify the length of the resulting PCR products. Gene expression was normalized to the geometric mean of ubiquitin-conjugating enzyme E2 I (Ube2i) expression levels.

**TABLE 2:** Table of qPCR primers used in the study.

	Forward primer	Reverse primer
Clec10a	GAGAAAAACCAAGAGGCTGGT	CTAAGGCCCAGGGAGAACA
Tgfb1	CCTGGAAAGGGCTCAACAC	CAGTTCTTCTCTGTGGAGCTGA
CD163	CTCAGCGTCTCTGCTGTCAC	GGCCAGTCTCAGTTCCTTCTT
Arg1	TTTCCTTGCCTGCTTCTTC	TCCTGTCTCCGTATTCAGCC
iNOS	GCAGAA TGTGACCATCATGG	ACAACCTTGGTGTTGAAGGC
CD86	TCCTCCAGCAGTGGGAAACA	TTTGTAGGTTTCGGGTATCCTTGC
Ube2i	AGCTACGGATGCTTTTCAAAGA	CAGAAGGATACACGTTTGGATGA

### Statistical Analysis

All graphs and statistics were performed in GraphPad Prism 6.0. The body asymmetry, Bederson’s score, and locomotor activity data were analyzed using Bonferroni’s *post hoc* test following two-way ANOVA. The infarction volume and qPCR results were analyzed using the two-tailed Student’s *t*-test. Two-way ANOVA and Tukey’s multiple comparisons *post hoc* test with the factors of “time” and “drug treatment” were used for the comparison of differences in ELISA results and parameters quantified from immunohistochemical staining. Values are presented as mean ± S.E.M. A statistically significant difference was defined as *p* < 0.05.

## Results

### Low-Dose VPA Treatment Does Not Reduce Infarction Volume, but Promotes Short-Term Behavioral Recovery in Rat Cortical Stroke Model

**FIGURE 1:**
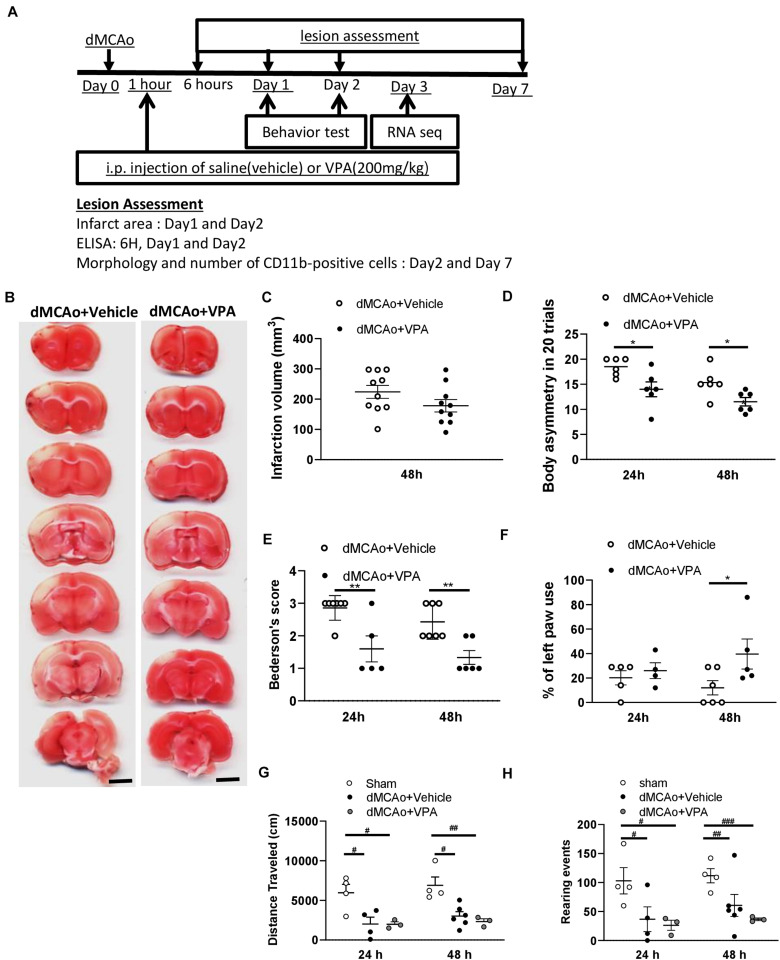
Post-stroke single injection of VPA (200 mg/kg) does not reduce infarct area, but promotes functional recovery. **(A)** Experimental timeline. The rats underwent dMCAo surgery, and they were divided into two groups randomly. One hour after reperfusion, the animals received an intraperitoneal injection of either VPA or vehicle once, and their behavioral functions were evaluated on day 1 and 2. Rats were sacrificed for analysis at different time points. **(B)** A representative image showing infarct area stained with TTC in a series of coronal sections derived from these two groups of rats killed at 48 h after ischemia. **(C)** Quantified results of infarct volumes derived from animals killed at 48 h after the onset of dMCAo. **(D,E)** Effects of VPA (*n* = 7), vehicle (*n* = 8) on body asymmetry **(D)**, Bederson’s neurologic test score **(E)**, *p < 0.05, **p < 0.01 indicate comparison with vehicle with Bonferroni’s *post hoc* test following two-way ANOVA. **(F)** Forepaw use bias of the rats was assessed using the cylinder test on day 1 and 2 after dMCAo. *p = 0.039 by Bonferroni’s multiple comparisons test, following two-way ANOVA. **(G,H)** Effects of VPA (*n* = 5), vehicle (n = 6), and no treatment (*n* = 4) on vertical **(G)** and horizontal **(H)** activity measured for 30 min on day 1 and 2. *p < 0.05, **p < 0.01; ^#^*p* < 0.05, ^##^*p* < 0.01, ^###^*p* < 0.001 by Bonferroni’s multiple comparisons test, following one-way ANOVA. Scale bar: 5000 μm. The data represent mean ± SEM.

In contrast with the results reported by prior studies, a single injection of the dose of VPA (200 mg/kg) had no effect on infarct volume measured at 48 h after stroke (Figures 1A–C). However, the treatment produced a rapid recovery of function, as seen from significantly reduced body asymmetry and Bederson’s scores at 24 h post-stroke (Figures 1D,E). At 48 h, the rats injected with VPA also exhibited substantial reversal of injury-induced behavioral deficits in terms of body asymmetry, Bederson’s neurological deficits, and the cylinder tests compared to the control group of vehicle-injected rats (Figures 1D–F). Spontaneous motor activity, as another indicator of hastened recovery, did not differ between the treatment groups (Figures 1G,H). These results clearly indicate that the low-dose VPA treatment significantly promoted functional recovery without reducing the size of the ischemic lesions.

### VPA Decreases the Production of IL-6, but Not TNF-α or IL-1β in the Peri-Infarct Cortex

**FIGURE 2:**
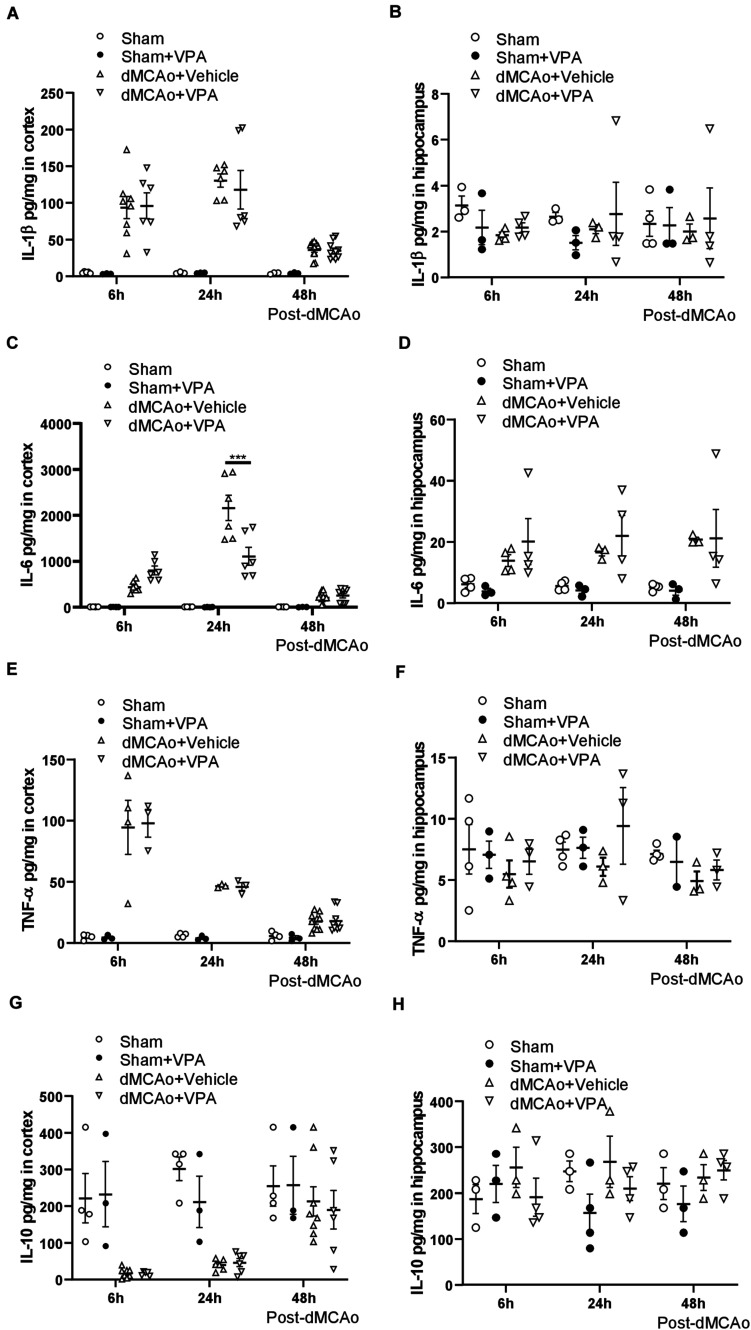
Valproic acid suppresses IL-6 production in the peri-infarct cortex at 24 h after dMCAo surgery. **(A,B)** IL-1β concentrations in the peri-infarct cortex **(A)** and ipsilateral hippocampus **(B)** were measured by ELISA. **(C,D)** TNF-α concentrations in the peri-infarct cortex **(C)** and ipsilateral hippocampus **(D)** were measured by ELISA. **(E,F)** IL-6 concentrations in the peri-infarct cortex **(E)** and ipsilateral hippocampus **(F)** were measured by ELISA. **(G,H)** IL-10 concentrations in the peri-infarct cortex **(G)** and ipsilateral hippocampus **(H)** were measured by ELISA. ***p < 0.001 indicates comparison with the dMCAo + vehicle group with two-way ANOVA, Bonferroni’s *post hoc* test. The data represent mean ± SEM.

Since the levels of pro-inflammatory cytokines in experimental stroke have significant effects on infarction evolution (Lambertsen et al., 2012; Suda et al., 2013), we first characterized the cytokine responses in the proximal (peri-infarct cortex) and distal (ipsilateral hippocampus) region after dMCAo-induced cortical stroke, and then tested the efficacy of post-stroke subcutaneous VPA treatment in adult rats. Obvious increases in IL-1β, TNF-α, and IL-6 were observed as soon as 6 h after dMCAo within the peri-infarct cortex, where they remained elevated for 2 days after dMCAo (Figures 2A,C,E). Of additional interest, down-regulated IL-10 was observed 6 h after dMCAo in the peri-infarct cortex, where it remained decreased until 24 h (Figure 2G). In the area distal from infarct cortex (hippocampus), there were no differences in the levels of IL-1β, TNF-α, and IL-10 between the sham and dMCAo groups at different time points (Figures 2B,F). However, increased IL-6 was observed in hippocampal brain homogenates 6 h after dMCAo, where it remained elevated until 2 days (Figure 2D). These results suggest that dMCAo-induced up-regulation of IL-6 is not only involved in the region proximal to the infarct cortex, but also in the region distal to it (hippocampus).

To determine whether post-stroke VPA treatment is involved in inflammatory responses, we measured changes in the levels of pro- and anti-inflammatory cytokines in the peri-infarct cortex and hippocampal brain homogenates at 6, 24, and 48 h after dMCAo. The administration of VPA resulted in a significant decrease in IL-6 within the peri-infarct cortex at 24 h after dMCAo (Figure 2C), but not in the ipsilateral hippocampal area (Figure 2D). Meanwhile, the levels of IL-1, TNF-α, and IL-10 within the peri-infarct cortex and ipsilateral hippocampus exhibited no statistically significant differences between the two groups at different time points (Figures 2B,F,H), implying that post-stroke VPA treatment only suppresses the up-regulation of glial activation-induced IL-6 in the peri-infarct environment.

### Post-Insult VPA Administration Reduces the Number of CD11b-Positive Cells on Day 7

**FIGURE 3:**
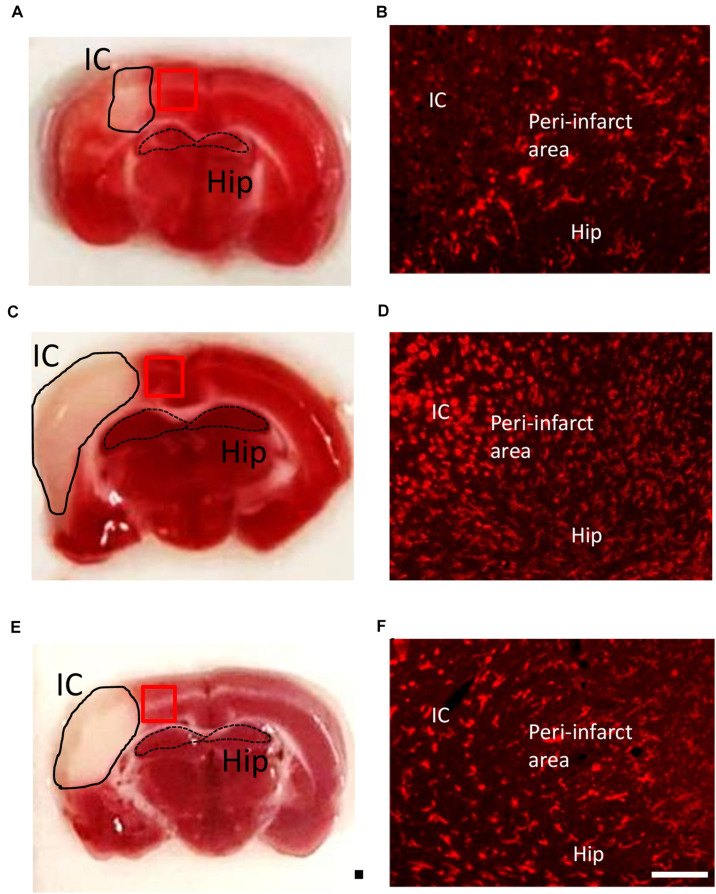
Time course of microglia/macrophage activation after cortical stroke. Representative images of TTC staining and immunostaining of all microglia/macrophages (CD11b) from ischemic core (IC), peri-infarct area, and hippocampus (Hip) coronal sections at 1 **(A,B)**, 2 **(C,D)**, and 7 **(E,F)** days after 90-min dMCAo in rats. The morphological changes and increasing number of microglia/macrophages were obviously found at 2 days after ischemia. Scale bar is 50 μm (immunofluorescence of CD11b) and 5000 μm (TTC staining).

**FIGURE 4:**
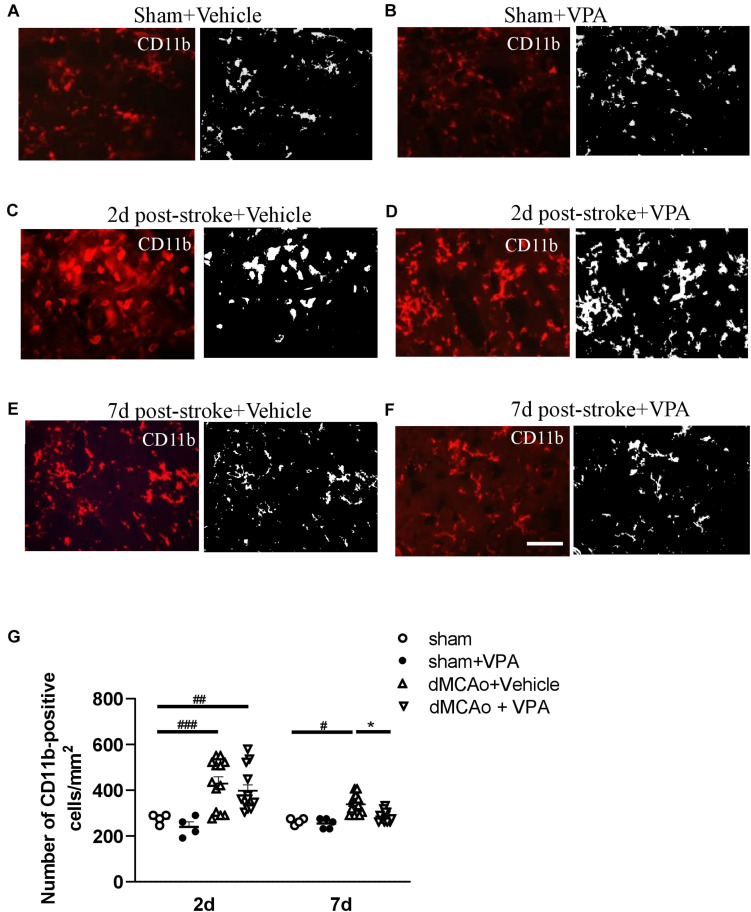
Post-stroke intraperitoneal injection of VPA decreases the number of CD11b-positive cells in the peri-infarct cortex. **(A–F)** Representative photomicrographs of anti-CD11b immunofluorescent staining of the sham-operated cortex in vehicle-treated rats **(A)**, sham-operated cortex in VPA-treated rats **(B)**, peri-infarct cortex at 2 days after 90-min dMCAo in vehicle-treated rats **(C)**, peri-infarct cortex at 2 days after 90-min dMCAo in VPA-treated rats **(D)**, peri-infarct cortex at 7 days after 90-min dMCAo in vehicle-treated rats **(E)**, and peri-infarct cortex at 7 days after 90-min dMCAo in VPA-treated rats **(F)**. **(G)** Quantitation of CD11b-positive cells in the peri-infarct cortex at different time points showing accumulation of activated microglia/macrophages in the peri-infarct cortex at 2 and 7 days after ischemia. Original photomicrographs were subjected to a series of uniform Image J plugin protocols prior to conversion to binary images; binary images were then analyzed to calculate the number of CD11b-positive cells. #*p* < 0.05, ##*p* < 0.01, and ###*p* < 0.001 indicate statistical difference between peri-infarct cortex and sham-operated cortex at each time point, Bonferroni’s multiple comparisons test, following one-way ANOVA. *p < 0.05 indicates comparison with the dMCAo + vehicle group with two-way ANOVA, Bonferroni’s *post hoc* test. Five tissue sections per rat were used for the analysis (*n* = 8–10). Scale bar: 20 μm. The data represent mean ± SEM.

Valproic acid has been demonstrated to exhibit a neuroprotective property in a rat model of dMCAo by promoting neuronal survival as well as exerting anti-inflammatory effects, which would contribute to a reduction in microglial cell numbers (Suda et al., 2013). However, how post-stroke VPA treatment modulates the time course of microglia/macrophage activation in the peri-infarct area has not yet been investigated. The infarct was already well advanced at 6 h after dMCAo, reached a maximum volume by 48 h, and had contracted greatly by 7 days based on the volume of tissue exhibiting pale staining with TTC (Figures 3A,C,E). Thus, we first characterized the time course of CD11b-positive microglia/macrophages in the dMCAo-induced ischemic cortex. At 6 h post-stroke, few CD11b-positive microglia/macrophages with ramification were present in the peri-infarct region (Figure 3B). Microglia/macrophage activation peaked on day 2 post-stroke in the ischemic cortex, when the infarct core and peri-infarct zone were filled with CD11b^+^ cells presenting with an amoeboid shape (Figure 3D). At 7 days post-stroke, CD11b-positive cells were obvious evident in the peri-infarct zone, but the morphology of microglia/macrophages with ramification of cell processes seemed to have recovered partially (Figure 3F). VPA treatment did not obviously reduce the number of CD11b-positive microglia/macrophages in sham group (Figures 4A,B,G) and on day 2 post-stroke (Figures 4C,D,G), but significantly decreased the CD11b-positive cell number in the peri-infarct zone at day 7 post-stroke (Figures 4E–G). These results suggested that the low-dose VPA treatment has delayed effects on the recruitment of microglia into ischemic cortex.

### VPA Treatment Regulates Morphologic Responses of CD11b-Positive Cells After Ischemic Brain Injury

**FIGURE 5:**
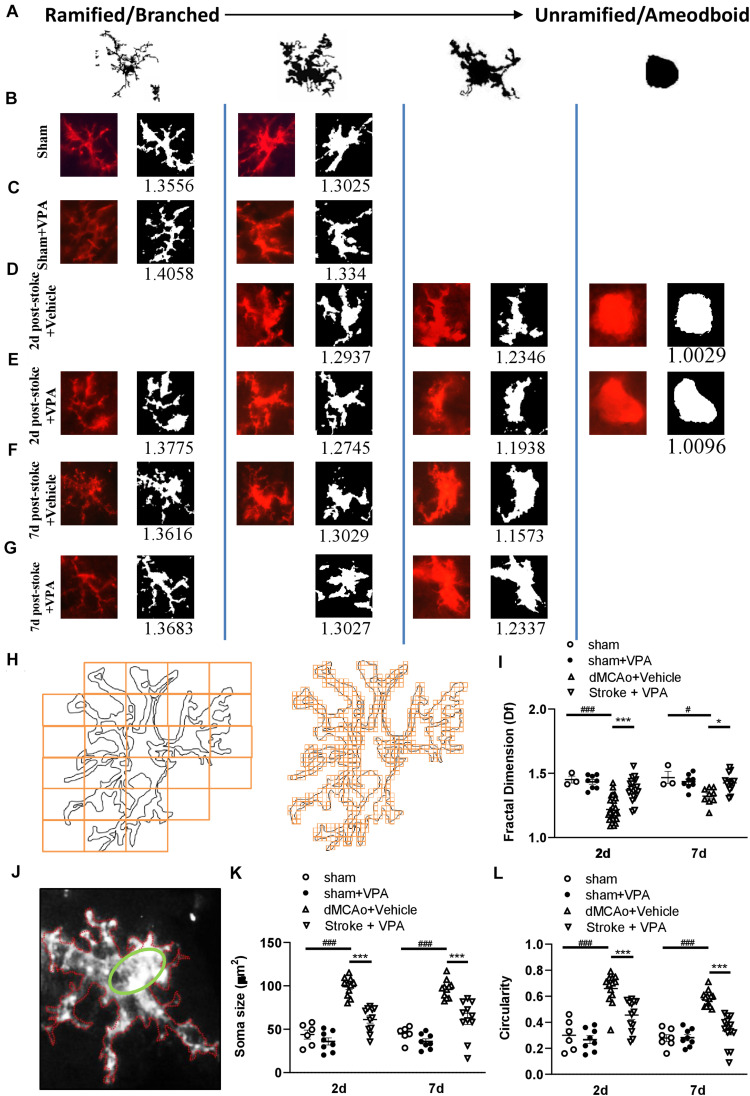
Complexity analysis of CD11b-positive cell morphologies in the peri-infarct cortex with/without VPA administration. **(A)** Microglia/macrophages are morphologically dynamic cells able to change form from highly ramified to a completely amoeboid-like shape lacking processes. This transition can be very rapid under pathological conditions. The forms illustrated here represent snapshots of a transformation that is reversible at every time point, with variation with each form shown. **(B–G)** Complexity analysis of microglia/macrophages in CD11b-stained tissue. The process to prepare photomicrographs for complexity analysis: Original photomicrographs were subjected to a series of uniform ImageJ plugin protocols prior to conversion to binary images. Binary images were then analyzed by using FracLac for ImageJ, which quantifies single cell complexity (fractal dimension, fD). The calculated Df of the cell is shown below its binary image. Representative images of morphological changes in CD11b-staining cells within the sham-operated cortex in vehicle-treated rats **(B)**, sham-operated cortex in VPA-treated rats **(C)**, peri-infarct cortex at 2 days after 90-min dMCAo in vehicle-treated rats **(D)**, peri-infarct cortex at 2 days after 90-min dMCAo in VPA-treated rats **(E)**, peri-infarct cortex at 7 days after 90-min dMCAo in vehicle-treated rats **(F)**, and peri-infarct at 7 days after 90-min dMCAo in VPA-treated rats **(G)**. **(H)** Illustration of FracLac box counting method to derive fractal dimension calculations of a CD11b-positive cell outline. Shape detail is quantified as scale increase, represented by orange boxes. Box counting equation is summarized in Table 1. **(I)** Summary data and statistical analysis of fractal dimension at 2 and 7 days after ischemia. Fractal dimension was decreased in the peri-infarct cortex compared with the sham-operated cortex at 2 and 7 days post-stroke. However, VPA treatment restored the fractal dimension of CD11b-positive cells in the peri-infarct cortex at 2 days and 7 days post-stroke. All *post hoc* analyses are reported in the figure (2 days: ###*p* < 0.001 vs. sham and dMCAo + vehicle; ***p < 0.001 vs. dMCAo + vehicle and dMCAo + VPA; 7 days: #*p* < 0.05 vs. sham and dMCAo + vehicle; *p < 0.05 vs. dMCAo + vehicle and dMCAo + VPA). **(J)** The schemes illustrate graphically the calculation base for the circularity index for the manual analysis. Q22. **(K,L)** Soma size and circularity index were increased in the peri-infarct cortex compared with the sham-operated cortex at 2 and 7 days post-stroke. However, VPA treatment decreased the soma size and circularity index of CD11b-positive cells in the peri-infarct cortex at 2 and 7 days post-stroke. All *post hoc* analyses are reported in the figure (2 days: ###*p* < 0.001 vs. sham and dMCAo + vehicle; ***p < 0.001 vs. dMCAo + vehicle and dMCAo + VPA; 7 days: ###*p* < 0.001 vs. sham and dMCAo + vehicle; ***p < 0.001 vs. dMCAo + vehicle and dMCAo + VPA). Twenty-five to thirty cells per region of *n* = 4 rats in each group.

To further characterize the morphological responses of the microglia, the fractal dimensions and cell circularity of CD11b-immunolabeled cells were measured in separate sections from the same rats at 2 and 7 days post-stroke. Examples of CD11b-positive microglia (made binary and outlined) in the peri-infarct cortex with/without VPA administration are shown in Figure 5. The application of FracLac for Image J to microglia outlines resulted in fractal dimensions that ranged from 1.0029 to 1.4058 (available range is 1–2), with the lowest occurring in the peri-infarction region at day 2 post-stroke and the highest in the intact cortex with sham surgery (Figures 5B,D,I). There was no difference in the fractal dimensions of CD11b-immunolabeled cells in the intact cortex with or without VPA administration (Figures 5C,I). However, two-way ANOVA analysis showed that VPA treatment could increase the fractal dimensions of CD11b-labeled microglia/macrophages at 2 and 7 days post-stroke, compared with the saline group (Figures 5D–G,I). The above results suggest that microglial complexity is unchanged from sham in the impact region with or without VPA application, whereas, in the peri-infarct region, cell complexity was lower at 2 and 7 days post-stroke and was recovered close to the sham condition through VPA therapy. Using manual analysis, we investigated an additional measure of microglial morphology related to cell circularity: the circularity index. The soma size and circularity index results of the CD11b-labeled cells in the sham group were similar to those of the CD11b-labeled cells in the VPA-treated group (Figures 5K,L). However, the soma size as well as circularity index was maximally increased at 48 h post-stroke, and remained similarly elevated by 7 days (Figures 5K,L). The administration of VPA caused a significant reduction in the soma size and circularity index of CD11b-positive cells compared with the vehicle-treated group when assessed at 2 and 7 days after stroke (Figures 5K,L). Based on qualitative observations of the CD11b-labeled cell complexity, soma size and circularity, it was suggested that VPA administration could be implicated in microglia activation in the peri-infarct region.

### VPA Suppresses the Level of Galectin-3 in the Peri-Infarct Cortex and LPS-Treated Microglia

**FIGURE 6:**
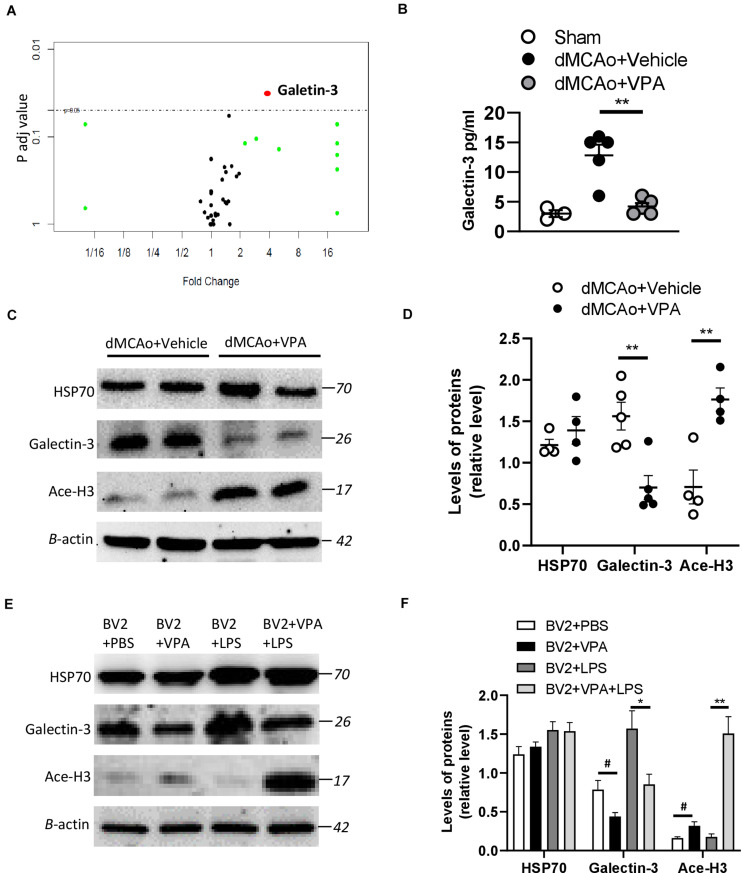
Effects of VPA treatment on galectin-3 expression in the peri-infarct cortex and BV2 cells. **(A)** Volcano plot comparing the fold changes and adjusted p-values of 67 biomarkers in which each point represents a biomarker. **(B)** VPA suppresses galectin-3 production in the peri-infarct cortex of rats with ischemic brain injury. Galectin-3 concentrations in the peri-infarct cortex at 2 days after dMCAo surgery were measured by ELISA (*n* = 5). **p < 0.01, compared with the vehicle-treated group. **(C)** Protein levels of HSP70, acetylated H3 (Ace-H3), galectin-3, and β-actin were determined by western blot analysis. **(D)** Quantified results of HSP70, Ace-H3, and galectin-3. Each column and vertical bar represents the mean ± SEM of 5 animals. **P < 0.01, Student’s *t*-test. **(E)** BV2 cells were activated using LPS (1 μg/ml) and, simultaneously, in the presence or absence of VPA as indicated. After 6 h of treatment, the protein levels of HSP70, acetylated H3 (Ace-H3), galectin-3, and β-actin were determined by western blot analysis. **(F)** VPA treatment enhances the levels of Ace-H3, but suppresses galectin-3 production in the BV2 cells with/without LPS exposure. #*p* < 0.05 vs. control. *p < 0.05 and **p < 0.01 indicate comparison with LPS-treated BV2 cells with Student’s *t*-test. The data represent mean ± SEM.

It is widely accepted that epigenetic processes occur during ischemic stroke (Stanzione et al., 2020). HDAC inhibitors are known to promote a phenotypic shift in microglia and subsequently exert neuroprotective effects in animal models of neurodegenerative diseases (Li S. et al., 2019; Li et al., 2020). VPA has been shown to exhibit anti-inflammatory properties by suppressing the number of activated microglia in stroke rats (Suda et al., 2013). However, it is much less clear whether VPA ameliorates the activation of microglia/macrophages through the regulation of HDACs or the modulation of certain protein expression levels in these cells. Therefore, we adopted the antibody array analysis of 67 proteins in sham-operated, vehicle-treated, and VPA-treated ipsilateral cortex 2 days after dMCAo (Table 3). Only proteins with fold change > 2 or <0.25 and which were significantly different (adjusted *P* < 0.05) were included (Figure 6A). The level of galectin-3 was significantly higher in the vehicle-treated stroke rats compared with the sham-operated rats (Figure 6B). However, VPA treatment significantly suppressed the level of galetin-3 in the ischemic cortex compared with the vehicle-treated group cortex (Figure 6B). Western blotting showed that the galectin-3 protein level was remarkably reduced in the VPA-treated peri-infarct cortex (Figures 6C,D), consistent with the results obtained with the protein array analysis. In previous studies, the VPA dose used in animal studies to control seizures, 300 mg/kg, was demonstrated to induce an increase in acetylated histone H3 levels and to up-regulate HSP70 levels in the brain. In our study, the dosage of VPA, revised down to 200 mg/kg, also increased acetylated histone H3 levels in the peri-infarct cortex compared with vehicle-treated group cortex (Figures 6C,D). However, there was no difference in the levels of HSP70 between the two groups (Figures 6C,D). Since galectin-3 is expressed mainly in microglia/macrophages and up-regulated when activated, we next determined the epigenetic mechanism whereby VPA may regulate galectin-3 production in these cells. BV2 cells were challenged with 1 μg/ml LPS in the presence or absence of 1.6 Mm VPA for 6 h. Acetylated histone H3, galectin-3, and HSP70 expressions in the BV2 cells were subsequently assessed. As presented in Figure 6E, the addition of VPA resulted in an increase over the baseline acetylated histone H3 level but decreased galectin-3 expression compared with untreated control. In addition, when cells were treated with LPS in the presence of VPA, acetylated histone H3 levels were significantly increased when compared with those receiving LPS treatment alone (Figure 6F). In contrast, administration with VPA was found to significantly decrease galectin-3 expression in LPS-treated cells as compared to LPS treatment alone (Figure 6F). However, the treatment of VPA had no effect on HSP70 expression under the condition of exposing BV-2 cells with or without LPS (Figures 6E,F). These results suggest that VPA, by promoting histone acetylation on H3, inhibits galectin-3 production in the microglia/macrophages under the baseline condition or in response to injurious stimuli.

**TABLE 3:** Comparison on 67 biomarkers between dMCAo + vehicle and dMCAo + VPA groups.

Biomarker	dMCAo + VPA	dMCAo + vehicle	FoldChange	statistic	p.value	FDR	entrez_id
Galectin-3	4.01(3.2, 6.18)	15.43(5.14, 15.77)	3.850125136	Wilcoxon *W* = 2	0.003175	0.031042	83781
Adiponectin	2.21 ± 1.92	7.46 ± 3.27	3.368981525	*t* = –3.0969	0.019277	0.531042	246253
IL-1 ra	0(0, 0)	21.98(0, 75.63)	2198.554233	Wilcoxon *W* = 2.5	0.02537	0.531042	60582
IL-6	7.52(0, 22.56)	0(0, 1.73)	0.001327755	Wilcoxon *W* = 22	0.044909	0.531042	24498
FGF-BP	301.74 ± 135.3	460.75 ± 66.35	1.526962995	*t* = –2.3594	0.057642	0.531042	64535
IL-1 R6	225.24(0, 3432.54)	0(0, 0)	4.44E-05	Wilcoxon *W* = 20	0.072006	0.531042	171106
*P*-Cadherin	0(0, 0)	10.43(0, 22.32)	1044.471407	Wilcoxon *W* = 5	0.072006	0.531042	116777
TREM-1	0(0, 0)	4.49(0, 10.61)	449.8794155	Wilcoxon W = 5	0.072006	0.531042	301229
SCF	3.77 ± 2.71	11.03 ± 7.76	2.928412671	*t* = –1.976	0.105526	0.623708	60427
JAM-A	7.58 ± 7.42	16.97 ± 9.35	2.237719561	*t* = –1.7577	0.118792	0.623708	116479
IL-2 R alpha	0(0, 1.8)	1.21(0, 43.35)	121.5922312	Wilcoxon *W* = 5	0.118797	0.623708	25704
HGF	26.28(0, 48.32)	132.12(0, 180.73)	5.026618741	Wilcoxon *W* = 5	0.138792	0.623708	24446
Erythropoietin	0(0, 173.89)	67(0, 257.54)	6700.768421	Wilcoxon *W* = 5.5	0.161238	0.623708	24335
CD48	0(0, 0)	0(0, 13.61)	1	Wilcoxon *W* = 7.5	0.179712	0.623708	245962
CINC-2	0(0, 1.29)	0(0, 0)	1	Wilcoxon *W* = 17.5	0.179712	0.623708	171551
gp130	0(0, 0)	0(0, 204.37)	1	Wilcoxon *W* = 7.5	0.179712	0.623708	25205
RAGE	0(0, 93.74)	0(0, 0)	1	Wilcoxon *W* = 17.5	0.179712	0.623708	81722
Neuropilin-1	916.22 ± 718.22	1491.07 ± 633.66	1.627402803	*t* = –1.342	0.216978	0.690058	246331
Prolactin	49.11(2.2, 104.24)	67.37(45.9, 234.69)	1.371723009	Wilcoxon *W* = 6	0.222222	0.690058	24683
Prolactin R	0(0, 394.66)	199.46(0, 860.2)	19946.8758	Wilcoxon *W* = 6.5	0.235861	0.695791	24684
CTACK	42601.63 ± 25019.88	60571.41 ± 20800.69	1.421809662	*t* = –1.2349	0.253013	0.706855	362505
CNTF	5.56 ± 6.14	10.8 ± 7.51	1.940993959	*t* = –1.206	0.263573	0.706855	25707
TIMP-1	1297.87 ± 1033.13	2371.34 ± 1771.85	1.827096283	*t* = –1.1703	0.283371	0.726908	116510
Notch-2	571.4(523.82, 1436.6)	744.09(542.62, 1286.46)	1.30221903	Wilcoxon *W* = 7	0.309524	0.742871	29492
Activin A	0(0, 0)	0(0, 560.91)	1	Wilcoxon *W* = 10	0.423711	0.742871	29200
b-NGF	0(0, 0)	0(0, 11.38)	1	Wilcoxon *W* = 10	0.423711	0.742871	310738
CINC-3	0(0, 0)	0(0, 0.85)	1	Wilcoxon *W* = 10	0.423711	0.742871	114105
EphA5	0(0, 0)	0(0, 58.11)	1	Wilcoxon *W* = 10	0.423711	0.742871	79208
IFNg	0(0, 0)	0(0, 0.03)	1	Wilcoxon *W* = 10	0.423711	0.742871	25712
IL-13	0(0, 1.14)	0(0, 0)	1	Wilcoxon *W* = 15	0.423711	0.742871	116553
LIX	0(0, 0.28)	0(0, 0)	1	Wilcoxon *W* = 15	0.423711	0.742871	60665
RANTES	0(0, 0)	0(0, 0.13)	1	Wilcoxon W = 10	0.423711	0.742871	81780
TIMP-2	0(0, 0)	0(0, 2.41)	1	Wilcoxon *W* = 10	0.423711	0.742871	29543
CINC-1	0(0, 6.32)	0(0, 2.52)	1	Wilcoxon *W* = 16	0.440686	0.742871	81503
MIP-1 alpha	0(0, 368.16)	0(0, 62.21)	1	Wilcoxon *W* = 16	0.440686	0.742871	25542
Gas 1	26.56 ± 18.14	35.3 ± 22.7	1.32868067	*t* = –0.672	0.521405	0.848722	683470
Fractalkine	1130.84(826.69, 2588.39)	879.85(779.54, 1895.16)	0.778050805	Wilcoxon *W* = 16	0.547619	0.848722	89808
MCP-1	79.53(24.26, 161.42)	119.8(29.26, 372.45)	1.506219934	Wilcoxon *W* = 9	0.547619	0.848722	24770
IL-4	0.06 ± 0.08	0.09 ± 0.05	1.401720264	t = -0.6091	0.56102	0.848722	287287
Notch-1	271.33 ± 150.9	283.29 ± 147.24	1.044064267	*t* = –0.1268	0.902226	1	25496
B7-1	0(0, 0.37)	0(0, 0.33)	1	Wilcoxon *W* = 13	1	1	25408
B7-2	0(0, 6.95)	0(0, 16.11)	1	Wilcoxon *W* = 12	1	1	56822
Fit-3 Ligand	0.66(0, 2.1)	0.65(0, 2.36)	0.979628446	Wilcoxon *W* = 13	1	1	1.04E + 08
IL-10	0(0, 11.54)	0(0, 3.16)	1	Wilcoxon *W* = 13	1	1	25325
Neuropilin-2	329.77(166.34, 1581.33)	348.82(46.11, 1051.13)	1.057751003	Wilcoxon *W* = 13	1	1	81527
TCK-1	789.04(504.99, 6581.18)	1229.66(217.51, 3259.77)	1.558425886	Wilcoxon *W* = 13	1	1	246358
TWEAK R	0(0, 14.54)	0(0, 51.69)	1	Wilcoxon *W* = 12.5	1	1	302965
4-1BB	0(0, 0)	0(0, 0)	1	Wilcoxon *W* = 12.5			500590
IL-17F	0(0, 0)	0(0, 0)	1	Wilcoxon W = 12.5			301291
IL-1a	0(0, 0)	0(0, 0)	1	Wilcoxon *W* = 12.5			24493
IL-2	0(0, 0)	0(0, 0)	1	Wilcoxon *W* = 12.5			116562
IL-3	0(0, 0)	0(0, 0)	1	Wilcoxon *W* = 12.5			24495
TIM-1	0(0, 0)	0(0, 0)	1	Wilcoxon *W* = 12.5			140898
TNFa	0(0, 0)	0(0, 0)	1	Wilcoxon *W* = 12.5			24835
VEGF	0(0, 0)	0(0, 0)	1	Wilcoxon *W* = 12.5			83785

### Differential Gene Expression in Peri-Infarct Cortex With or Without VPA Treatment

**FIGURE 7:**
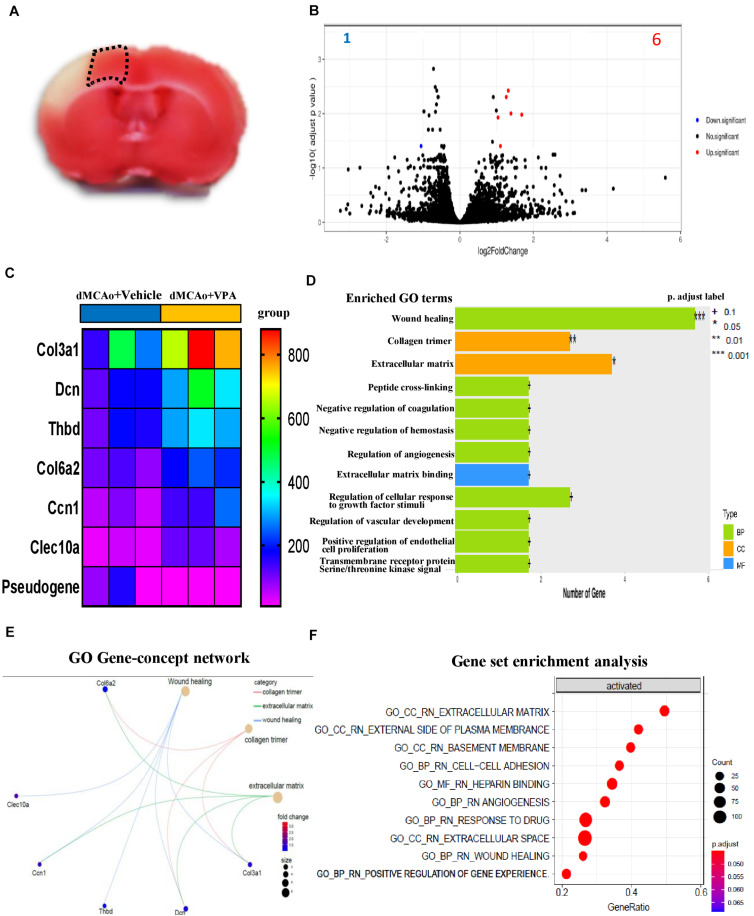
Post-stroke VPA treatment regulates gene expression in the peri-infarct cortex at 3 days after ischemic brain injury. **(A)** A representative image of the brain sections used for the RNA-sequencing experiment. **(B)** Volcano plot comparing the log2fold changes and adjusted *p*-values of 15446 gene expressions. The red dots indicate genes upregulated (log2fold change > 1, adjusted *p*-value < 0.05), the blue dots indicate genes downregulated (log2fold change < –1, adjusted *p*-value < 0.05), and the black dots indicate genes with no significant change between dMCAo + vehicle and dMCAo + VPA. **(C)** Heatmap plot of DEGs in peri-infarct cortex between dMCAo + vehicle and dMCAo + VPA. **(D)** GO biological processes, cellular component, and molecular function over-representation analysis based on 7 DEGs. **(E)** Molecular network plot connected using GO over-representation analysis following VPA treatment in a rat model of dMCAo. **(F)** GO gene set enrichment analysis of genes upregulated by VPA.

Since the low dose of VPA was implicated as having an effect on the level of galectin-3 and in the morphological changes of microglia in the peri-infarct cortex, we naturally hypothesized that VPA treatment would regulate genes involved in tissue repair, as well as the phenotypes of immune cells. Comparison of gene profiles between vehicle-treated and VPA-treated groups was focused on the peri-infarct cortex at 3 days post-stroke (Figure 7A). Genes were identified as differentially expressed genes (DEGs) only when the fold difference between two groups was greater than 2 and the adjusted *p*-values were lower than or equal to 0.05. Volcano plots that graphically highlighted the DEGs that were significantly up- (red) or down- (blue) regulated in response to VPA treatment and vehicle treatment in the stroke rats were generated (Figure 7B). In the VPA-treated group, seven transcripts were differentially expressed compared to the vehicle-treated group, among which one gene was downregulated and six genes were upregulated (Figure 7C). The one down-regulated gene was a pseudogene. The up-regulated genes were related to components of the extracellular matrix (collagen 3a1, collagen 6a2, decorin, and cellular communication network factor 1) and included genes which code for coagulation-related surface proteins on the endothelial cells (thrombomodulin) and macrophage galactose-type lectin on M2 microglia (C-type lectin domain containing 10A) (Figure 7C). These data revealed a prominent upregulation of gene expression related to extracellular matrix remodeling in the stroke rats that received VPA treatment.

### Gene Ontology (GO) Analysis From VPA- and Vehicle-Treated Ischemic Brains

To further reveal the functional categories of DEGs, gene ontology (GO) enrichment analysis was performed. GO enrichment analysis of DEGs (dMCAo + vehicle vs. dMCAo + VPA) identifies key biological processes, cellular components, and molecular functions. The top three significantly upregulated functional categories (adjusted *P*-value < 0.05) were enriched by DEGs in the VPA-treated group, with those DEGs being involved in wound healing, collagen trimmer and extracellular matrix functions (Figures 7D,E). Notably, the DEGs related to wound healing were also partially associated with collagen trimmer and extracellular matrix functions, suggesting that post-stroke VPA treatment might potentiate tissue remodeling in the peri-infarct cortex. Network-based GSEA (gene set enrichment analysis) was utilized to identify the functional associations amongst the genes affected by the VPA treatment (Figure 7F). Similar to the results obtained using enriched GO analysis, the top 10 functions of the up-regulated genes were associated with the extracellular space (adjusted *P*-value = 0.044), drug responses (adjusted *P*-value = 0.044), extracellular matrix (adjusted *P*-value = 0.044), positive regulation of gene expression (adjusted *P*-value = 0.044), basement membrane (adjusted *P*-value = 0.044), external side of the plasma membrane (adjusted *P*-value = 0.044), cell–cell adhesion (adjusted *P*-value = 0.046), wound healing (adjusted *P*-value = 0.046), heparin binding (adjusted *P*-value = 0.046), and angiogenesis (adjusted *P*-value = 0.046).

### Post-stroke VPA Treatment Regulates Gene Expression Profile of Activated Microglia

**FIGURE 8:**
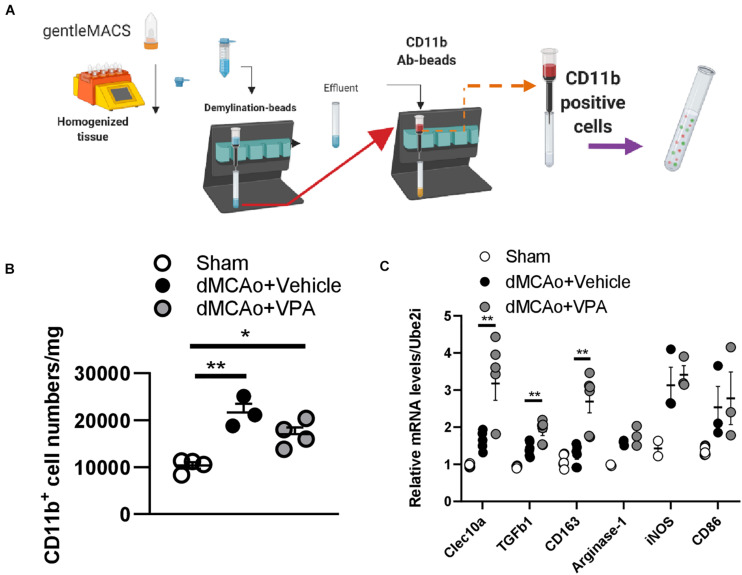
Post-stroke VPA treatment enhances gene expressions of the alternative activated M2 phenotypes. **(A)** Male rats (12 weeks old) were subjected to sham operation or dMCAo surgery with/without VPA treatment. Microglia/macrophages were purified from sham cortex and peri-infarct cortex at day 3 post-dMCAo using gentleMACs Dissociator and CD11b antibody-beads. **(B)** There were significant differences in the number of CD11b-expressing microglia/macrophages between the sham group and dMCAo groups. **(C)** qPCR analysis of gene expression of alternative activated M2 phenotype (*Clec10a*, *TGF*β*1, CD163*, and *Arginase-1*) and classical activated M1 phenotype (*iNOS* and *CD86*) in the CD11b-expressing microglia/macrophages of sham-operated, dMCAo + vehicle, and dMCAo + VPA groups. *P < 0.05, **P < 0.01, Student’s *t*-test. *N* = 3–4 per group. The data represent mean ± SEM.

Given that the C-type lectin domain containing 10A (*Clec10a*) was mainly expressed on the antigen-presenting cells (**APC**) and macrophages (Van Vliet et al., 2008), we wondered whether the upregulated *Clec10a* in VPA-treated peri-infarct cortex was also on the activated microglia. Therefore, we isolated CD11b-positive cells from the infarct area at day 3 post-stroke (Figure 8A), and then analyzed the mRNA level of *Clec10a* in these cells. Similar to the results obtained from immunofluorescence, VPA treatment did not obviously reduce the number of CD11b-positive microglia on day 3 post-stroke (Figure 8B). However, a significant increase in the mRNA level of *Clec10a* in the CD11b-positive microglia/macrophages was shown in the VPA-treated group (Figure 8C), suggesting that treatment with VPA increased *Clec10a* expression in the peri-infarct cortex and on activated microglia and macrophages. Since *Clec10a* has immunoregulatory properties and is induced on M2 microglia in neurological autoimmune disorders (Ilarregui et al., 2019), we next wanted to explore whether VPA could regulate the phenotypes or gene expression profiles of microglia. For additional evaluations of the status of microglia/macrophages, the mRNA levels of selected markers of the cytotoxic M1 phenotype (*iNOS* and *CD86*) and the cytoprotective M2 phenotype (*TGF*β*1, CD163, Arginase-1*) were analyzed in these CD11b^+^ cells. Our results indicated that VPA treatment does not change the expressions of *iNOS* or *CD86*, genes expressed in cytotoxic M1-type microglia (Figure 8C). In turn, the analysis of markers of M2 phenotype revealed a significant increase in the mRNA levels of *TGF*β*1* and *CD163* but not *Arginase-1* in the CD11b^+^ microglia in response to VPA exposure (Figure 8C), implying that VPA treatment did not cause collective up-regulation of M2-type marker genes in the peri-infarct cortex. Taken together, these findings indicate that post-stroke VPA treatment not only stimulates extracellular matrix remodeling, but also alters the morphological responses and gene expressions of activated microglia in the peri-infarct cortex.

## Discussion

This study provides evidence that a low dose of VPA post-stroke can improve the recovery of aspects of neurological function in rats, and that this effect of VPA is associated with the suppression of peri-infarct activity in microglia, including activity related to morphological changes and density. This attenuation of microglial activity may involve the suppression of galectin-3 expression and the upregulation of extracellular matrix remodeling in the peri-infarct cortex.

Accumulating evidence supports the notion that histone hypoacetylation and transcriptional dysfunction are involved in a large number of neurodegenerative conditions (Hu et al., 2017). Although postischemic treatment with a clinically relevant dose of VPA, specifically, 300 mg/kg, has been shown to reduce dMCAo-induced brain infraction, BBB disruption, and brain edema (Ren et al., 2004; Wang et al., 2011), this dose is too high for humans. As with many antiepileptic drugs, there are a number of adverse consequences associated with the use of VPA. Among these, valproate encephalopathy is a dose-related syndrome that can occur due to high VPA concentrations, particularly in new patients (Hamer et al., 2000). Moreover, VPA-associated pancytopenia and coagulopathy have been shown to be dose-dependent side effects (Attilakos et al., 2007). Furthermore, the teratogenicity of VPA limits its use in woman of childbearing age (Gotlib et al., 2017). In this study, we reduced the dose to 200 mg/kg of VPA and tested the therapeutic effects of this dose in rats. The results indicated that a single injection of VPA at a dose of 200 mg/kg in stroke rats did not reduce infarct volume but did significantly accelerate the reversal of behavioral deficits in rats with cerebral ischemic injury. The improvements of forepaw function and body symmetry occurred in the absence of changes in infarct volume and are broadly consistent with reports of improved outcomes without changes in infarct volume following VPA treatment in several studies (Lee et al., 2014). Thus, the findings of the present study suggest that cellular changes critical for recovery are initiated in the early stages after infract formation, and that they can be modulated by VPA treatment.

It has become increasingly that clear brain injury following ischemia is highly associated with the inflammatory response, which involves the infiltration of mononuclear phagocytes and activated microglia (Glass et al., 2010; Shi et al., 2011). Inflammatory cytokines (IL-1β, TNF-α, and IL-6) produced by peri-infarct microglia/macrophages would modulate tissue injury and have profound effects on infarct evolution (Lambertsen et al., 2012). In the past literature, VPA was demonstrated to inhibit TNF-α and IL-6 production induced by LPS in THP-1 cells, and this inhibition was linked to the suppression of NF-κB activation (Ichiyama et al., 2000; Mairuae and Cheepsunthorn, 2018). In animal studies of global brain ischemia, VPA further suppressed IL-1β production in the hippocampus (Xuan et al., 2012). Studies of cellular sources of TNF-α and IL-1β after MCAo have looked specifically at microglia and macrophages. However, IL-6 is upregulated in microglia and cortical neurons in stroke rats, and increases of IL-6 are more pronounced in the case of gray matter lesions (Eriksson et al., 1999; Suzuki et al., 1999). Based on our results, a single injection of VPA at a dose of 200 mg/kg was shown to suppress the up-regulation of IL-6 in cortex at 24 h following dMCAo, suggesting that low-dose VPA therapy might both alleviate neuronal damage and dampen microglial activation in the early phase of cerebral ischemic injury.

The single injection of a low dose of VPA after stroke did not produce significant differences in the number of CD11b-positive microglia/macrophages within the peri-infarct cortex within the first 2 days. Thus, our findings suggest that the low dose of VPA did not produce gross alterations in CD11b-positive cell recruitment to the peri-infarct tissue in the early phase. However, in other studies regarding the effect of VPA on microglia/macrophage distribution, VPA was shown to greatly limit microglial density in the injured area (Xuan et al., 2012; Suda et al., 2013). This difference in response to focal ischemia compared with that in the present study is probably explained, at least in part, because the higher dose of VPA in those other studies produced decreases in cell loss and infarct size in those earlier investigations of stroke, and such protective effects can secondarily reduce microglial responses to ischemic damage.

Apart from the changes in microglial distribution in response to ischemic damage, ramified microglia would transform to amoeboid morphology and resume phagocytotic activity, which exacerbate neuroinflammatory responses (Sierra et al., 2016; Norris and Kipnis, 2019). It follows that microglia morphology, if quantified using sensitive methods, could provide insight into the neuropathology of distinct regions. Using two quantitative assessments of microglia complexity and circularity, we report that the microglia morphology was different from that in sham cortex in the peri-infarct cortex at 2 and 7 days post-stroke. In the peri-infarct region, most of the microglia became amoeboid or had increased circularity, while ramification in shape or higher complexity was less observed. One striking finding of our results is that the low-dose VPA treatment produced significant differences in the complexity and circularity of CD11b-positive microglia within 7 days compared with vehicle treatment, indicating that VPA greatly limits changes in microglial morphology after dMCAo. Simultaneously, the low-dose VPA treatment was shown to not only enhance the levels of acetylated histone H3 protein but also to suppress the levels of galectin-3 in the peri-infarct cortex. Since galectin-3 is predominantly expressed in the microglia of the adult rodent brain (Lalancette-Hebert et al., 2012; Reichert and Rotshenker, 2019), we further argue that VPA treatment could suppress the LPS-upregulated galectin-3 levels in BV2 cells. It has been shown that galectin-3 could control microglia morphology by regulating the cytoskeleton (Hoyos et al., 2014; Reichert and Rotshenker, 2019). Thus, amoeboid microglia are rich in galectin-3 and display productive phagocytosis. Moreover, galectin-3 is required for microglia-mediated brain inflammation (Nomura et al., 2017; Siew et al., 2019), while the suppression of galectin-3 ameliorates microglia-mediated pathogenesis by decreasing NFκB activation (Siew et al., 2019). Interestingly, NFκB motifs have been identified in the promotor region of galectin-3 (Hsu et al., 1996), implying that NFκB and galectin-3 might be regulated in a positive feed-forward loop in microglia. In our study, VPA was shown for the first time to promote a morphological shift of peri-infarct microglia from the amoeboid to the ramified form, and that might be associated with galectin-3 suppression in the peri-infarct cortex or microglia. Although previous studies have found some epigenetic pathways wherein the chromatin remodeling proteins contribute to galectin-3 induction (Li Z. et al., 2019), we have not yet examined whether VPA regulating the level of galectin-3 is directly dependent on the epigenetic action or upregulation of the acetylation of NFκB p65 caused by the decrease of HDAC3 activity (Wang et al., 2011).

An increasing number of studies have demonstrated that VPA treatment could directly up-regulate genes associated with neuronal proliferation, differentiation, and neurotransmission, while down-regulating genes related to cell death and inflammation (Nikolian et al., 2018). Such changes may play roles in the benefits provided by VPA treatment following injury. In our study, RNA sequencing further revealed that VPA treatment exhibits a strong trend of upregulation of genes related to extracellular matrix remodeling in the peri-infarct cortex. Collagen 3a1, collagen 6a2, decorin, and cellular communication network factor 1, all of which are known to be part of the extracellular matrix, serve essential functions involved in the regulation of cellular processes and providing a permissive microenvironment to promote tissue repair (Nelimarkka et al., 2001; Davis and Senger, 2005; Kubota and Takigawa, 2007; Jarvelainen et al., 2015; Gregorio et al., 2018; Ucar and Humpel, 2018). Moreover, the provisional extracellular matrix serves as a pliable scaffold wherein mechanical guidance forces are established among endothelial cells, thereby providing critical support for vascular endothelium (Davis and Senger, 2005). Thrombomodulin is a membrane protein mainly expressed by endothelial cells. It is part of the anticoagulant protein C system, in which thrombomodulin binds with thrombin and promotes the cleavage of protein C and thrombin activatable fibrinolysis inhibitor, thereby inhibiting coagulation (Wenzel et al., 2014). Additionally, it interferes with inflammation, stabilizes barrier function, and promotes angiogenesis under pathological conditions (Watanabe-Kusunoki et al., 2020). *Clec10a* is a member of the C-type lectin receptor family and is expressed by myeloid APC, such as dendritic cells and macrophages (Ilarregui et al., 2019). Notably, *Clec10a* has also been shown to be upregulated on M2 microglia, which are involved in the processes of extracellular matrix reconstruction and tissue repair in the injured area. GSEA using GO also demonstrated a pattern of increased extracellular matrix, cell-cell adhesion, angiogenesis, and wound healing. Thus, the fact that these changes correlate with activated tissue repair and extracellular matrix suggests that VPA may help to restore normal functions.

In the present study, the analysis of VPA-induced changes to gene expression in the peri-infarct CD11b-positive cells at early time points revealed an up-regulation of the expression of *Clec10a, TGF*β*1*, and *CD163*, but not *Arginase-1.* These genes are expressed in M2 phenotype microglia. The effect of VPA in terms of altering the expression of markers of alternative, anti-inflammatory microglia (M2 phenotype) is in agreement with what has been reported after spinal cord injury (Chen et al., 2018), but unlike what the authors of that report proposed, our data suggest that, during the early phase of cerebral ischemic injury, VPA did not decrease the expression of M1-associated markers (iNOS, CD86). This discrepancy implies that the single injection with a low dose of VPA only stimulated certain subtypes of alternatively activated microglia during pathology progression. Primarily M2 phenotype microglia with high expression of *Arginase-1* contribute to axon regeneration through anti-inflammatory effects in neurodegenerative diseases (Cherry et al., 2014). CD163 is a phagocytic marker of microglia/macrophages that functions as a membrane-bound scavenger receptor for cleaning extracellular haptoglobin-hemoglobin and has an immunoregulatory property associated with the resolution phase of inflammation (Etzerodt and Moestrup, 2013). *TGF*β*1* has been recently shown to potentiate an adaptive activation of microglia to accelerate wound healing (Taylor et al., 2017). Collectively, these genes of the M2 phenotype are essential for repair processes, and their high expression in our study might reflect peri-infarct tissue repair in response to VPA treatment.

There were several limitations to the present study. First, although our data indicated that 200 mg/kg of VPA still exhibits a therapeutic effect in ischemic brain injury, we did not measure the kinetics of VPA in rats, and the dose we used on the rats in this study cannot be extrapolated to a human-equivalent dose. Second, our data demonstrated that post-stroke VPA treatment could suppress the upregulation of galectin-3, which is required for resident microglia activation in response to ischemic injury. However, galectin-3 knockout microglia failed to activate and proliferate, which was further associated with significant increases in the size of the ischemic lesions (Lalancette-Hebert et al., 2012). Thus, it is still necessary to elucidate the time courses of galectin-3 expression and function in microglia under pathological conditions. Finally, we did not have gene expression data from a sham group, so it is unclear if the effects of VPA on gene expression are restorative or *de novo*. Additional studies are already underway to fill in many of these gaps.

## Conclusion

We found that a single injection of VPA following dMCAo was able to accelerate functional recovery in rats. Mitigating microglia activation through the suppression of upregulated galectin-3 and altering the gene profiles of these cells could influence extracellular matrix reconstruction and contribute to the improved recovery.

## Data Availability Statement

The datasets presented in this study can be found in online repositories. The names of the repository/repositories and accession number(s) can be found below: NCBI BioProject database, PRJNA694497.

## Ethics Statement

The animal study was reviewed and approved by National Defense Medical Center’s Animal Center. Written informed consent was obtained from the owners for the participation of their animals in this study.

## Author Contributions

T-TK, Y-HC, and K-YT: conception and design. T-TK, VW, and K-YT: performing experiments, data analysis, and manuscript writing. T-TK, J-SW, Y-HC, K-YT: planning experiments, data analysis, and manuscript revision. All authors contributed to the article and approved the submitted version.

## Conflict of Interest

The authors declare that the research was conducted in the absence of any commercial or financial relationships that could be construed as a potential conflict of interest.

## Footnotes

1 https://www.ncbi.nlm.nih.gov/sra

## Abbreviations

AAALAC International: Association for Assessment and Accreditation of Laboratory Animal Care International
APC: antigen-presenting cells
BBB: Blood-brain barrier
CCA: common carotid artery
cDNA: complementary DNA
CI: circularity index
DEGs: differentially expressed genes
dMCA: distal middle cerebral artery
dMCAo: distal middle cerebral artery occlusion
DMEM: Dulbecco’s modified Eagle’s medium
dscDNA: double-stranded cDNA
ELISAs: enzyme-linked immunosorbent assays
FBS: fetal bovine serum
GSEA: gene set enrichment analysis
HDAC: histone deacetylase
HSP70: heat shock protein 70
IACUC: Institutional Animal Care and Use Committee
Iba1: ionized calcium binding adaptor molecule 1
LPS: lipopolysaccharide
MACS: magnetic activated cell sorting
qPCR: quantitative PCR
RLE: Relative Log Expression
TMM: trimmed mean of *M*-values
TTC: 2,3,5-triphenyltetrazolium chloride
VPA: valproic acid
WGCNA: weighted gene co-expression network analysis.
