# Supplementary figures and images for: Post-stroke Delivery of Valproic Acid Promotes Functional Recovery and Differentially Modifies Responses of Peri-Infarct Microglia
Source: Front Mol Neurosci. 2021 May 28;14:639145. doi: 10.3389/fnmol.2021.639145 (PMC8194695; doi:10.3389/fnmol.2021.639145)

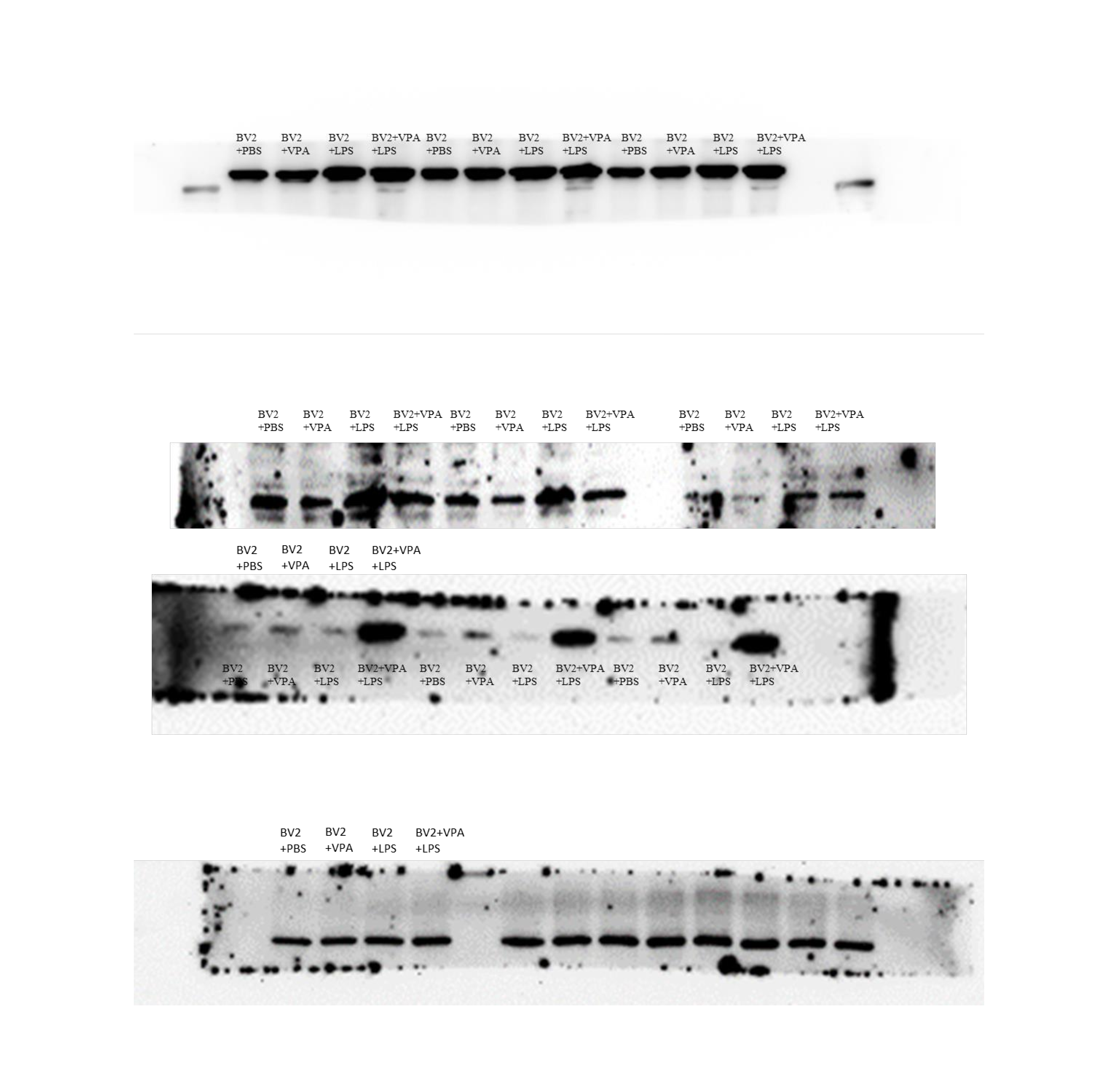

Supplement: Supplementary file 1 [file Data_Sheet_1.ZIP › Supplementary Figure3.tiff]
